# Multidisciplinary Treatment With Hepatic Arterial Infusion Chemotherapy, Radiotherapy, and Immunotherapy for Advanced Hepatocellular Carcinoma With Major Vascular Invasion: Prospective Registry Protocol

**DOI:** 10.2196/82992

**Published:** 2026-03-31

**Authors:** Yoshiko Doi, Hiroshi Aikata, Yumi Kosaka, Takashi Nakahara, Michiyo Kodama, Masashi Hieda, Masakazu Hashimoto, Hideki Nakahara, Ippei Takahashi, Hideaki Kakizawa, Nami Mori, Keiji Tsuji, Nobuki Imano, Yuji Murakami, Saki Sueda, Tomokazu Kawaoka, Masataka Tsuge, Shiro Oka

**Affiliations:** 1 Department of Radiation Oncology, Hiroshima Prefectural Hospital, Hiroshima, Japan; 2 Department of Gastroenterology and Hepatology, Hiroshima Prefectural Hospital, 1-5-54, Ujinakanda, Minami-ku, Hiroshima, 734-8530, Japan, +81 82 254 1818; 3 Department of Medical Oncology, Hiroshima Prefectural Hospital, Hiroshima, Japan; 4 Department of Diagnostic Radiology, Hiroshima Prefectural Hospital, Hiroshima, Japan; 5 Department of Gastroenterological Surgery, Hiroshima Prefectural Hospital, Hiroshima, Japan; 6 Department of Radiation Oncology, Hiroshima Red Cross Hospital and Atomic-bomb Survivors Hospital, Hiroshima, Japan; 7 Department of Diagnostic Radiology, Hiroshima Red Cross Hospital and Atomic-bomb Survivors Hospital, Hiroshima, Japan; 8 Department of Gastroenterology and Hepatology, Hiroshima Red Cross Hospital and Atomic-bomb Survivors Hospital, Hiroshima, Japan; 9 Department of Radiation Oncology, Hiroshima University Hospital, Hiroshima, Japan; 10 Department of Gastroenterology, Hiroshima University Hospital, Hiroshima, Japan; 11 Liver Center, Hiroshima University Hospital, Hiroshima, Japan

**Keywords:** hepatocellular carcinoma, macroscopic vascular invasion, hepatic arterial infusion chemotherapy, radiotherapy, immunotherapy, prospective registry study

## Abstract

**Background:**

Systemic therapy, including immune checkpoint inhibitors, has improved survival in advanced hepatocellular carcinoma (HCC); however, its efficacy remains limited in patients with macroscopic vascular invasion (MVI), a subgroup with an extremely poor prognosis. Although combining immunotherapy with local treatments such as hepatic arterial infusion chemotherapy (HAIC) and radiation therapy (RT) is considered a promising approach, robust supportive evidence from routine clinical practice is lacking.

**Objective:**

This study aims to evaluate the safety and therapeutic effectiveness of a multidisciplinary treatment strategy involving RT after HAIC, followed by immunotherapy, in patients with MVI-positive HCC, using real-world clinical data from Japan.

**Methods:**

This is a prospective, multicenter registry study conducted at 3 hospitals in Hiroshima Prefecture, Japan. Eligible patients will have unresectable MVI-positive HCC confirmed by dynamic computed tomography. The treatment protocol follows a standardized sequence: 1 session of HAIC (cisplatin), RT targeting the MVI site (25 Gy in 5 fractions), and subsequent systemic immunotherapy. The primary end point is safety, which will be evaluated using the Common Terminology Criteria for Adverse Events (version 5.0). The secondary end points include progression-free survival at 12 and 24 weeks, tumor response, median progression-free survival, overall survival, and objective response rate at 12 and 24 weeks, assessed according to the Response Evaluation Criteria in Solid Tumors criteria. Data will be collected prospectively and analyzed according to the intention-to-treat principle.

**Results:**

Patient enrollment began in March 2025, and data collection and analysis are ongoing as participants continue to be followed.

**Conclusions:**

This prospective registry study will generate real-world evidence on the safety and effectiveness of a multidisciplinary strategy combining HAIC, RT, and immunotherapy in patients with MVI-positive HCC. Given that all components are covered under Japan’s national health insurance, this approach could be readily implemented in clinical practice and may inform future treatment guidelines for MVI-positive HCC.

## Introduction

In the treatment of unresectable, locally advanced hepatocellular carcinoma (HCC), multikinase inhibitors such as sorafenib and lenvatinib were long regarded as standard first-line therapies, as established by the SHARP (Sorafenib HCC Assessment Randomized Protocol) and REFLECT (Study of Lenvatinib vs Sorafenib in First-Line Treatment of Patients With Unresectable Hepatocellular Carcinoma) trials [1,2]. However, recent landmark phase III trials—IMbrave150 (atezolizumab plus bevacizumab) [3], HIMALAYA (durvalumab plus tremelimumab) [4], and CheckMate 9DW (nivolumab plus ipilimumab) [5]—have demonstrated that immune checkpoint inhibitor–based combination regimens significantly improve overall survival (OS), resulting in a paradigm shift in the management of advanced HCC [6,7].

Despite these advances, immune checkpoint inhibitor–based regimens have shown limited benefit in patients with macroscopic vascular invasion (MVI), one of the most prognostically unfavorable subgroups. In IMbrave150, atezolizumab plus bevacizumab significantly improved OS in high-risk patients—including those with the main portal vein tumor thrombus, bile duct invasion, or ≥50% liver involvement—yet the median OS in this subgroup was only 7.6 months [8]. Moreover, the HIMALAYA and CheckMate 9DW trials excluded patients with the main portal vein tumor thrombus, leaving a lack of robust clinical evidence for this population.

Before the advent of immunotherapy, several studies demonstrated that combining locoregional therapies—such as hepatic arterial infusion chemotherapy (HAIC) and radiation therapy (RT)—with systemic agents could improve outcomes in MVI-positive HCC [9-13]. In a phase III trial, He et al [9] showed that HAIC using the FOLFOX regimen (folinic acid, fluorouracil, and oxaliplatin) plus sorafenib yielded a markedly higher overall response rate (ORR; 40.8% vs 2.46%; *P*<.001) and longer progression-free survival (PFS; 7.03 vs 2.6 months; *P*<.001) than sorafenib monotherapy for HCC with portal vein invasion. A Japanese prospective study also demonstrated improved survival with HAIC using the cisplatin (CDDP) regimen plus sorafenib vs sorafenib alone in patients with major portal vein tumor thrombus (median OS 11.4 vs 6.5 months) [10]. Furthermore, in a large-scale multicenter retrospective study conducted in Japan using propensity score matching, HAIC using the CDDP was associated with better survival outcomes than sorafenib monotherapy in MVI-positive patients without extrahepatic lesions (median OS 11.4 vs 6.5 months; *P*=.02) [11]. RT also has been shown to improve survival in patients with MVI-positive HCC, as demonstrated in a randomized clinical trial [12]. In particular, Kosaka et al [13] reported an ORR of 19.6%, a thrombus response rate of 51.0%, and a median OS of 19.8 months for the combination therapy of HAIC using the CDDP and RT in cases of MVI-positive HCC.

Since the advent of immunotherapy, combination treatments of HAIC with immunotherapy [14-17] or RT with immunotherapy [18] have been increasingly implemented for MVI-positive HCC, particularly in Asia. In both approaches, the addition of immunotherapy has tended to enhance therapeutic efficacy. This may be attributable to the induction of immunogenic cell death by HAIC or radiotherapy through tumor necrosis, thereby potentiating the effects of immunotherapy [19,20]. Furthermore, combining all 3 modalities—HAIC, RT, and immunotherapy—may also offer promising therapeutic potential. In patients with MVI-positive HCC, RT may contribute to local tumor control within the portal vein, thereby helping to preserve hepatic blood flow and liver function. Short-course hypofractionated RT can facilitate timely initiation of subsequent systemic therapy while minimizing treatment-related delays. A case series by Yamaoka et al [21] described 6 patients treated with atezolizumab plus bevacizumab following HAIC and RT, with 4 achieving survivals exceeding the previously reported median OS values. These findings suggest that multimodal strategies targeting both intrahepatic tumor burden and vascular invasion may offer meaningful clinical benefits for this challenging patient population.

## Methods

### Study Design and Setting

**Figure 1.**
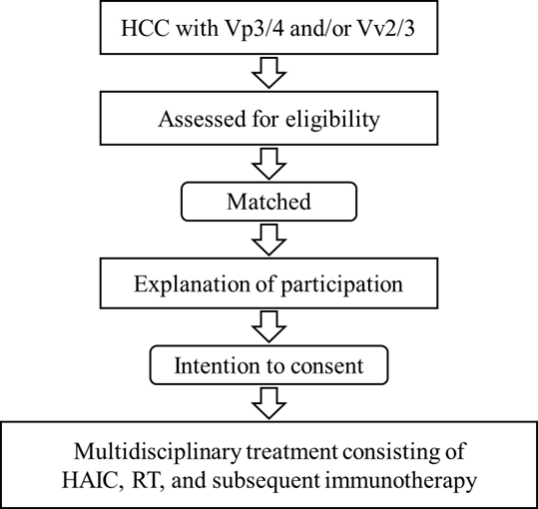
Study flow of the prospective observational registry. Patients with hepatocellular carcinoma (HCC) with a tumor thrombus in the first-order branch or main trunk of the portal vein (Vp3/4) and/or a tumor thrombus in the inferior vena cava or hepatic veins (Vv2/3) are assessed for eligibility. Eligible patients receive an explanation of study participation and provide informed consent. Enrolled patients then undergo multidisciplinary treatment consisting of hepatic arterial infusion chemotherapy (HAIC), radiation therapy (RT), and subsequent immunotherapy according to routine clinical practice. Patients are followed longitudinally for safety and efficacy outcomes.

This study is a multicenter, prospective observational registry designed to collect real-world data on patients with MVI-positive HCC who are treated according to routine clinical practice at participating institutions in Hiroshima Prefecture, Japan (Hiroshima Prefectural Hospital, Hiroshima University Hospital, and Hiroshima Red Cross Hospital & Atomic-bomb Survivors Hospital). The treatment sequence of HAIC, RT, and subsequent immunotherapy reflects standard clinical practice at these institutions and is not mandated by the study protocol. The protocol does not direct or assign specific treatments; rather, it prospectively records treatment decisions made by the treating physicians. The described timing between treatment modalities represents typical clinical practice and target intervals, allowing for physician discretion based on individual patient conditions. Patient enrollment began in March 2025. On the basis of the sample size estimation, a total of 38 consecutive eligible patients will be enrolled, with longitudinal data collection until treatment discontinuation or death. No randomization or intervention assignment will be performed, and all treatment decisions will follow routine clinical practice. An overview of the study design is shown in Figure 1.

### Ethical Considerations

This study was conducted in accordance with the ethical principles of the Declaration of Helsinki and was approved by the institutional review boards (IRBs) of all participating institutions. The study protocol, including data collection and informed consent procedures, was reviewed and approved by the Hiroshima Prefectural Hospital IRB (approval 202503‐1), the Hiroshima University Hospital IRB (approval e2024-0174-02), and the Hiroshima Red Cross Hospital & Atomic-bomb Survivors Hospital IRB (approval P7-10-1). Written informed consent will be obtained from all participants prior to enrollment. Participants will not receive any financial compensation for participation in this study.

All study data will be managed using institution-managed secure storage systems at each participating site in accordance with institutional policies and applicable data protection regulations. Patient data will be collected as part of routine clinical practice and deidentified prior to analysis. Access to study data will be restricted to authorized study personnel based on their roles, and data handling, storage, and transfer between participating institutions will be conducted using secure, institution-approved methods in compliance with ethical and legal requirements.

All monitoring activities will be conducted in compliance with institutional policies, relevant regulations, and the principles of Good Clinical Practice, ensuring that participant safety and data integrity are maintained throughout the study period.

### Patient Population

Eligible participants are patients with unresectable, locally advanced HCC with MVI who are scheduled to receive multidisciplinary treatment consisting of HAIC, RT, and subsequent immunotherapy. MVI is defined radiologically as a tumor thrombus in the first-order branch or main trunk of the portal vein (Vp3/4) or in the inferior vena cava or hepatic veins (Vv2/3) based on dynamic contrast-enhanced computed tomography (CT). Key inclusion and exclusion criteria are summarized in Textbox 1.

Textbox 1. **Key inclusion and exclusion criteria.**

**Inclusion criteria**

- Hepatocellular carcinoma with macroscopic vascular invasion diagnosed by dynamic computed tomography
  - Vp3/Vp4: portal vein (first-order branches or main trunk)
  - Vv2/Vv3: tumor thrombus in inferior vena cava or hepatic veins (left, middle, or right)
- Age ≥18 years
- Eastern Cooperative Oncology Group performance status: 0‐2
- Chronic hepatitis or liver cirrhosis (Child-Pugh class A or B with score≤7)
- Written informed consent obtained

**Exclusion criteria**

- History of prior radiotherapy overlapping the planned field
- Unsuitable for immunotherapy
- Uncontrolled ascites or hepatic encephalopathy
- Active gastric or duodenal ulcers or untreated esophageal or gastric varices or hemorrhagic ulcers
- Severe infections or comorbidities (excluding chronic hepatitis or liver cirrhosis)
- Psychiatric disorders interfering with study participation (per investigator judgment)
- Active double cancers (concurrent malignancies)
- Contraindication for computed tomography imaging (eg, allergy to iodinated contrast)
- No informed consent provided
- Any other reason deemed inappropriate by the attending physician

### Treatment Protocol

#### Overview

**Figure 2.**
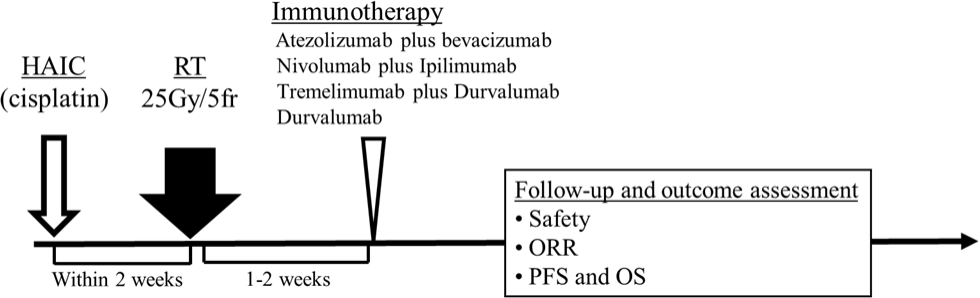
Outcome assessment during follow-up. Patients are followed longitudinally after multidisciplinary treatment to evaluate safety and efficacy outcomes. Safety is assessed throughout the treatment and follow-up period, while efficacy outcomes include objective response rate (ORR), progression-free survival (PFS), and overall survival (OS). CDDPia: cisplatin intra-arterial infusion; HAIC: hepatic arterial infusion chemotherapy; RT: radiation therapy.

A schematic of the study flow and treatment sequence is shown in Figure 2. All eligible patients will receive 1 session of HAIC followed by RT targeting the MVI site and then systemic immunotherapy. The interval between treatments will generally be 1 to 2 weeks. Modifications to the sequence or addition of other therapies may be made at the treating physician’s discretion based on therapeutic response and clinical condition.

#### HAIC Procedure

HAIC will be performed promptly after diagnosis in clinically appropriate patients with adequate liver function (Child-Pugh class A or B7), acceptable renal function, and no contraindications to CDDP-based chemotherapy. Single-dose CDDP was selected for HAIC because it is the only agent approved and reimbursed for this indication within the Japanese public health insurance system and has demonstrated favorable efficacy and safety in patients with HCC with MVI in a previous study [22].

Catheter insertion will generally be performed via the femoral artery using the Seldinger technique. Abdominal angiography will confirm tumor staining and feeding arteries. A catheter will then be inserted into the feeding artery, and CDDP (65 mg/m²) will be infused over 20 to 40 minutes, with earlier termination permitted if sufficient tumor perfusion is achieved. If the estimated glomerular filtration rate is <60 mL/min/1.73 m², the CDDP dose will be reduced by 75%. When CDDP is infused into only part of the liver, the dose will be adjusted according to the proportion of tumor volume in the liver based on CT imaging.

To prevent CDDP-induced nephrotoxicity, patients will receive adequate intravenous hydration before and after infusion. Patients will be hospitalized for observation for at least 24 hours after infusion, with monitoring of vital signs, urine output, and laboratory tests to assess renal and hepatic function. Prophylactic antibiotics will be administered according to institutional protocols.

#### Radiotherapy

RT will be initiated preferably within 2 weeks after completion of HAIC. While specific technical approaches will be left to each institution’s policy, two points will be standardized across all sites: (1) the irradiated target volume should primarily encompass the intravascular portion of the tumor while including, whenever feasible, the adjacent intrahepatic lesion; and (2) the prescribed dose should be 25 Gy in 5 fractions.

Under the supervision of a radiation oncologist at each institution, an appropriate margin of 5 to 20 mm will be added to the target volume to account for respiratory motion and setup error, aiming for a homogeneous dose distribution within the target while minimizing exposure to surrounding organs at risk. Dose constraints for organs at risk will follow institutional protocols, with typical limits including a mean dose to the normal liver (whole liver excluding the tumor) of <13 Gy, a maximum dose to the stomach or duodenum of <30 Gy, and a maximum spinal cord dose of <25 Gy.

Patients will be immobilized using customized vacuum cushions or equivalent devices to ensure reproducibility, with respiratory motion management (eg, breath-hold) used throughout treatment with each facility’s policies. Either 3D conformal radiotherapy or intensity-modulated radiotherapy may be used. Image-guided radiotherapy, typically with daily cone-beam CT, is recommended for treatment delivery.

All treatment plans will be generated using a treatment planning system capable of Monte Carlo–equivalent dose calculation. Pretreatment plan quality assurance (QA) will be performed according to institutional standards, and daily machine QA will be conducted in accordance with international recommendations. Adaptive replanning will be considered if significant anatomical changes or tumor regression are observed during treatment.

#### Immunotherapy

Systemic immunotherapy will be initiated preferably within 2 weeks after the completion of RT, provided that adequate hematologic, hepatic, and renal function is confirmed. Regimens may include atezolizumab (1200 mg IV) plus bevacizumab (15 mg/kg IV) every 3 weeks; durvalumab (1500 mg IV) plus tremelimumab (300 mg IV once), followed by durvalumab monotherapy every 4 weeks (STRIDE [single tremelimumab regular interval durvalumab] regimen); or nivolumab (80 mg IV) plus ipilimumab (3 mg/kg IV) every 3 weeks, followed by nivolumab (240 mg IV) every 2 weeks or nivolumab (480 mg IV) every 4 weeks.

The choice of regimen will be determined at the discretion of the treating physician, taking into account patient comorbidities, previous treatment history, baseline organ function, and potential contraindications. In patients for whom combination immunotherapy is considered unsuitable, durvalumab monotherapy (1500 mg IV every 4 weeks) may be considered as an alternative option.

All regimens will adhere to recommendations outlined in established guidelines for systemic therapy in HCC, including the American Society of Clinical Oncology guideline for advanced HCC [23] and the Japan Society of Hepatology’s 2021 clinical practice guidelines [6], as well as the European Society for Medical Oncology Clinical Practice Guideline [24].

Treatment will continue until radiologically confirmed disease progression, unacceptable toxicity, or loss of clinical benefit, whichever occurs first. Temporary dose delays, reductions, or regimen modifications will be permitted according to protocol-specific criteria and clinical judgment. All modifications, interruptions, or discontinuations will be documented in detail, including the reason and subsequent management.

Patients will undergo routine laboratory monitoring, including complete blood count, liver and renal function tests, and coagulation parameters, at each treatment cycle. Imaging assessments (dynamic contrast-enhanced CT or magnetic resonance imaging [MRI]) will be performed in accordance with the study’s assessment schedule to evaluate tumor response and detect potential treatment-related complications, such as immune-related adverse events (irAEs). Prompt initiation of appropriate management strategies (eg, corticosteroids or immunosuppressants) will be undertaken for clinically significant irAEs, following current consensus recommendations [25-27].

### Data Collection

Collected data will include the following:

- Patient background—age, sex, Eastern Cooperative Oncology Group performance status, comorbidities, prior cancer history, and prior HCC treatments
- Baseline liver function—etiology of liver disease, presence of ascites or hepatic encephalopathy, Child-Pugh class, and relevant laboratory values
- HAIC—procedure date, infusion duration, and CDDP dose
- RT—start and completion dates, technique, treatment planning system, dose calculation algorithm, respiratory motion management, dose parameters, and dose-volume histogram metrics
- Immunotherapy—regimen, dosage, initiation date, and treatment duration
- Laboratory trends—serial laboratory test results during the treatment course
- Additional therapies—details of any subsequent locoregional or systemic treatments administered during follow-up

### Assessment Schedule

Clinical and laboratory assessments will be performed at baseline, at HAIC administration, at the start of RT, and at the initiation of immunotherapy. Adverse events (AEs) will be graded according to the Common Terminology Criteria for Adverse Events (CTCAE; version 5.0) and assessed at each visit or whenever clinically indicated. Specific laboratory monitoring for irAEs, including liver function, renal function, thyroid function, and blood glucose, will be conducted regularly. Imaging (contrast-enhanced CT, MRI, or ultrasound) will be performed at baseline, at 6 weeks and 12 weeks after starting immunotherapy, and every 2 months thereafter (+1 or −1 week), with tumor response evaluated according to Response Evaluation Criteria in Solid Tumors (RECIST; version 1.1) and/or modified RECIST (mRECIST) criteria.

### Safety Assessment

Safety outcomes will be evaluated descriptively for the overall study population. AEs will be summarized according to immunotherapy regimen where feasible. As this study is a prospective observational registry reflecting real-world clinical practice, no formal comparative analyses between immunotherapy regimens are planned.

### Sample Size Estimation

The primary objective of this study is to evaluate the safety of a multidisciplinary treatment strategy. Accordingly, the planned sample size was determined based on feasibility considerations and the desired precision of descriptive estimates rather than formal hypothesis testing. With an anticipated enrollment of 38 patients, the incidence of major AEs and the ORR can be estimated with an acceptable level of precision, as reflected by the width of the corresponding CIs. No formal hypothesis testing is planned, and all analyses will be descriptive and exploratory in nature.

### Statistical Analysis

All analyses will be performed using descriptive statistics to summarize patient characteristics, treatment delivery, and outcomes. Continuous variables will be presented as medians with IQRs or means with SDs, as appropriate. Categorical variables will be expressed as counts and percentages.

Time-to-event end points (PFS and OS) will be estimated using the Kaplan-Meier method, with median survival times and 95% CIs reported. Exploratory subgroup analyses may be performed using the log-rank test and Cox proportional hazards regression models, adjusting for baseline covariates such as liver function, tumor burden, and treatment regimen. Hazard ratios with 95% CIs will be calculated. In multicenter analyses, participating institutions may be included as stratification factors.

ORR will be calculated with exact binomial 95% CIs. Safety will be summarized by incidence, severity (CTCAE version 5.0), and relationship to study treatment, with results presented overall and stratified by treatment phase (HAIC, RT, and immunotherapy). The timing of onset, resolution, and reasons for treatment discontinuation due to AEs will also be reported.

Missing data will not be imputed; patients without an event at data cutoff will be censored at the date of last contact. Reasons for missing data will be recorded, and sensitivity analyses may be conducted under different missing data assumptions (eg, worst-case imputation for progression events).

Given the observational nature of the study, all analyses will be exploratory and hypothesis generating. No formal sample size calculation has been performed, and *P* values from statistical tests will be interpreted descriptively without adjustment for multiplicity. Statistical analyses will be conducted using the EZR (Saitama Medical Center, Jichi Medical University), a graphical user interface for R (version 3.4.1; The R Foundation for Statistical Computing).

### Monitoring and QA

No formal coordinating center has been established for this study. Instead, the principal investigator and participating investigators will jointly oversee all aspects of study conduct. Regular meetings (either in person or via teleconference) will be held to review study progress, verify the accuracy and completeness of collected data, and ensure adherence to the study protocol.

Any protocol deviations, data inconsistencies, or other study-related issues identified during these reviews will be documented in meeting minutes. Corrective actions will be determined through consensus among the investigators, and follow-up will be performed to confirm resolution.

### Protocol Deviations

All deviations from the approved protocol will be documented by the responsible investigator at each participating site, including the date, details, and reasons for the deviation. Given the investigator-driven nature of this study, no formal deviation classification system will be applied; however, any deviations judged by the investigators to potentially impact patient safety or data reliability will be discussed collectively among the study team.

Corrective actions will be decided through investigator consensus and implemented promptly. Records of all deviations and their resolution will be maintained securely by each investigator and shared within the study group as necessary. As this is not a formally regulated clinical trial, deviations will not be reported to regulatory authorities but will be transparently described in the final study report or publication when relevant.

### Dissemination Plan

The findings from this study will be disseminated through publication in peer-reviewed journals and presentations at national and international scientific meetings. While the raw dataset will not be made publicly available, anonymized summary data may be shared upon reasonable request and with investigator approval.

## Results

### End Points

#### Primary End Point

The primary end point is safety, defined as the incidence of treatment-related AEs graded according to the CTCAE (version 5.0), occurring during the treatment period after the last dose of any study treatment. Treatment-related AEs are those considered by the investigator to be possibly, probably, or definitely related to any component of the study regimen. AEs will be summarized descriptively for the overall cohort and, where feasible, stratified by immunotherapy regimen.

#### Secondary End Points

The secondary end point is PFS at 12 and 24 weeks, overall median PFS, OS, and ORR at 12 and 24 weeks. The ORR will be defined as the proportion of patients achieving a complete response or partial response as their best overall response, assessed according to the RECIST (version 1.1) and/or mRECIST for HCC, in which viable tumor is defined as uptake in the arterial phase of dynamic imaging. Tumor response will be evaluated by dynamic contrast-enhanced CT or MRI at baseline, at 6 and 12 weeks after initiation of immunotherapy, and every 2 months thereafter. In patients with measurable lesions at baseline, all target and nontarget lesions will be assessed, and any unmeasurable but evaluable lesions (eg, tumor thrombus) will be included in accordance with the RECIST (version 1.1) and/or mRECIST guidelines. PFS is defined as the time from initiation of treatment to the first documented disease progression per the RECIST (version 1.1) and/or mRECIST or death from any cause, whichever occurs first. OS is defined as the time from initiation of treatment to death from any cause. Patients without an event at the time of analysis will be censored at the date of the last disease assessment. Patients lost to follow-up will be censored at the date they were last known to be alive. Given the limited sample size, analyses of efficacy outcomes, including PFS and OS, will be descriptive and exploratory in nature, and no formal hypothesis testing is planned.

### Trial Status

As of March 2026, patient enrollment had already commenced, and data collection was actively ongoing. Participating investigators continue to recruit eligible patients and record study data in accordance with the protocol schedule. Patient enrollment began in March 2025, and data collection and analysis are ongoing as of March 2026.

## Discussion

The primary objective of this prospective registry study is to evaluate the safety and therapeutic effectiveness of a multidisciplinary treatment approach that combines locoregional therapies and immunotherapy in patients with MVI-positive HCC.

In Japan, both the incidence and mortality rates of HCC have declined over recent decades, largely owing to the decreasing prevalence of viral hepatitis. According to the 24th Nationwide Follow-up Survey Report on Primary Liver Cancer, the numbers of patients with Vp3/4 and Vv2/3 tumor thrombi in 2016 to 2017 were approximately 980 and 570, respectively, underscoring the relative rarity of these high-risk cases. Furthermore, the prevalence of MVI-positive HCC has continued to decrease since that period, making such cases even less common in current clinical practice. Because of this low and declining incidence, conducting adequately powered prospective randomized controlled trials in this population is challenging, and a real-world, prospective registry design was, therefore, considered the most feasible and appropriate approach to capture treatment patterns and outcomes in a sufficiently large cohort.

In this multidisciplinary treatment strategy, RT was intended to achieve local tumor control within the portal vein and to preserve portal venous blood flow, thereby maintaining liver function and eligibility for subsequent systemic therapy. Even modest tumor shrinkage may be clinically meaningful in patients with advanced HCC and major vascular invasion. A hypofractionated regimen of 25 Gy in 5 fractions in this study was selected to minimize treatment duration and allow timely initiation of immunotherapy, as longer radiotherapy courses or treatment-related toxicity could delay systemic therapy. This regimen represents a pragmatic balance between local disease control, safety, and uninterrupted transition to systemic therapy in real-world clinical practice.

A critical consideration is that MVI-positive HCC is associated with extremely poor prognosis, making the prompt initiation of aggressive treatment after diagnosis essential to maximize the potential for improved outcomes. Consequently, treatment strategies should prioritize modalities that are widely applicable and readily implementable in routine clinical settings. All components of this protocol—HAIC, RT, and immunotherapy—are established treatment methods in daily clinical practice in Japan, with minimal variation in availability or technical feasibility across institutions. Therefore, if this protocol demonstrates both safety and favorable efficacy, it could be rapidly adopted across many treatment centers, providing substantial clinical benefit for patients with difficult-to-treat MVI-positive HCC in Japan.

Recent reports suggest that multidisciplinary, team-based treatment may facilitate conversion to resectable status and improve long-term survival rates in patients with unresectable, MVI-positive HCC [28]. The sequential multidisciplinary collaboration strategy used in this study seeks to enhance therapeutic efficacy by leveraging the synergistic effects of each treatment modality. Furthermore, we anticipate that this approach will increase resectability and ultimately lead to better long-term outcomes.

Several limitations of this study should be acknowledged. As multiple immunotherapy regimens with different mechanisms of action and safety profiles are included, this study is not designed to distinguish regimen-specific safety signals, particularly given the limited sample size. This heterogeneity reflects real-world clinical practice in Japan, where several approved and reimbursed immunotherapy regimens with comparable efficacy are available and treatment selection is left to physician discretion. Therefore, safety findings should be interpreted descriptively and with caution.

In conclusion, this prospective registry study aims to generate valuable real-world evidence to inform treatment decisions for MVI-positive HCC and to contribute to the development of more effective therapeutic strategies in clinical practice.

## Abbreviations

AE: adverse event
CDDP: cisplatin
CT: computed tomography
CTCAE: Common Terminology Criteria for Adverse Events
FOLFOX: folinic acid, fluorouracil, and oxaliplatin
HAIC: hepatic arterial infusion chemotherapy
HCC: hepatocellular carcinoma
IRB: institutional review board
mRECIST: modified Response Evaluation Criteria in Solid Tumors
MRI: magnetic resonance imaging
MVI: macroscopic vascular invasion
ORR: objective response rate
OS: overall survival
PFS: progression-free survival
QA: quality assurance
RECIST: Response Evaluation Criteria in Solid Tumors
REFLECT: Study of Lenvatinib vs Sorafenib in First-Line Treatment of Patients With Unresectable Hepatocellular Carcinoma
RT: radiation therapy
SHARP: Sorafenib HCC Assessment Randomized Protocol
STRIDE: Single Tremelimumab Regular Interval Durvalumab
Vp3/4: a tumor thrombus in the first-order branch or main trunk of the portal vein
Vv2/3: a tumor thrombus in the inferior vena cava or hepatic veins
